# Cardiovascular disease prevention in women: are we up to date?

**Published:** 2011-06

**Authors:** Naomi Rapeport

**Affiliations:** Milpark Hospital, Johannesburg, South Africa

A pandemic of cardiovascular disease (CVD) is afflicting women. Heart disease is the leading cause of death in women in every major developed country and most emerging economies.^1^ Although it is often thought of as a disease of affluence, CVD mortality rates in women over the age of 60 years are more than double in low- and middle-income countries than in high-income countries.^2^

Much of the burden of this disease can be attenuated by addressing critical risk factors such as hypertension, type 2 diabetes mellitus (DM), dyslipidaemia, physical inactivity, tobacco use, overweight and obesity. These risk factors account for 63% of the deaths due to CVD and DM, and over three-quarters of the deaths from coronary heart disease (CHD).^3^ Tobacco use, overweight and obesity are currently more prevalent in middle- and high-income countries. However this situation may change, as it is projected that by 2030, almost 75% of tobaccorelated deaths will occur in low- and middle-income countries.^3^

In high-income countries, cardiovascular mortality rates in women have declined. This is secondary to modifications in risk behaviour, such as reduced tobacco use and increased physical activity, better management of hypertension and dyslipidaemia, and improved treatment of existing cardiovascular conditions.^4^ These benefits are not apparent in low- and lower middle-income countries where only a quarter of women with chronic heart disease receive treatment.^5^

In the USA, a high-income country, these positive trends are changing. CHD mortality rates in women aged 35 to 54 years are increasing, attributed to the obesity epidemic.^4^ Nearly two out of every three American women over 20 years of age are overweight or obese.^6^ This rise in obesity is a major contributor to the increased prevalence of DM, which has a direct impact on the overall risk of myocardial infarction (MI) and stroke.^7^ In some ethnic groups, there is a higher prevalence of certain risk factors, such as hypertension among African-American women and DM in Hispanics.^6^ African-American women have the highest CHD death rates and the highest overall CVD morbidity and mortality 6

In Africa, data from the Interheart Study showed that women of African ancestry presented with their first MI at a younger age than those from western Europe and North America (median age of 56 vs 68 and 64 years, respectively).^8^ In the Heart of Soweto study, women presented with CVD also in their fifties, and were slightly younger than the men (53 vs 55 years, *p* = 0.031).^9^ In this cohort, heart failure (HF) was the most common primary diagnosis. Few had coronary artery disease, but they had a high prevalence of cardiovascular risk factors, particularly hypertension and obesity.

While most of the morbidity and mortality from CVD occurs at older ages, exposure to these risk factors starts earlier in life, and therefore preventive interventions need to target younger women. The first women-specific clinical recommendations for the prevention of CVD were published in 1999, even though there were little gender-specific research data.^10^ Prior to this, it was advised that women be treated the same as men despite the exclusion of women from most clinical trials. Since the late 1990s, increasing numbers of women have participated in CVD studies, resulting in gender-specific analyses. Furthermore, major randomised, controlled clinical trials in women, such as the Women’s Health Initiative, have changed the practice of CVD prevention.^11^

In 2004, the Evidence-Based Guidelines for Cardiovascular Disease Prevention in Women were published.^12^ The 2004 guidelines confirmed that menopausal therapy [hormone-replacement therapy (HRT) and selective oestrogen-receptor modulators] was not a preventive treatment modality. It was given a class III status (i.e. not useful/effective and may cause harm) for both primary and secondary prevention of CVD. Oestrogen HRT had previously been advocated for all postmenopausal women with coronary and other vascular disease. To date this recommendation has remained unchanged. Other class III interventions that also remain unchanged include the use of antioxidant supplements such as vitamins E, C and beta-carotene, and folic acid with or without vitamin B_6_ and B_12_ supplementation. The routine use of aspirin in healthy women under 65 years of age is not recommended to prevent MI.

In 2004, the Global Risk Assessment was advocated to stratify patients’ CHD risk. It utilises the Framingham risk score, whereby, based on the level of risk, drug therapy for hypercholesterolaemia is advised, according to the National Cholesterol Education Panel Adult Treatment Panel III (NCEP ATP III) targets.

In the 2007 update of these guidelines, emphasis was placed on pre-clinical detection of disease to identify asymptomatic individuals at high risk who could benefit from early intervention.^13^ A new algorithm for risk classification was adopted that stratified women into three categories. Women at ‘high risk’ are those with documented CVD (such as established CHD, cerebrovascular disease, peripheral arterial disease or abdominal aortic aneurysm), DM, chronic kidney disease, or a 10-year predicted risk for CHD of 20% or more. ‘At-risk’ women have one or more major CVD risk factors [such as cigarette smoking, poor diet, physical inactivity, obesity (particularly central adiposity), family history of premature CVD (male relative below 55 years or female relative below 65 years), hypertension, dyslipidaemia, evidence of subclinical vascular disease (e.g. coronary calcification), metabolic syndrome, poor exercise capacity on treadmill test, and abnormal heart rate recovery after stopping exercise]. Women are regarded as ‘at optimal risk’ if they have no major CVD risk factors and engage in a healthy lifestyle.

Despite these guidelines, there are problems with risk stratification of women. There is a general underestimation of CHD risk, which has focused on short-term (10-year) risk and on MI and CHD death. Women with a high prevalence of subclinical disease are scored as low risk.^6^,^14^ In non-Caucasian populations there are problems of risk estimation, and in the elderly there is also an underestimation of risk. A woman aged 75 years with several risk factors will score below a 10% 10-year predicted risk for CHD.^15^,^16^ Few women qualify for lipid-lowering therapy for prevention of CHD.

In the most recent updated 2011 guidelines, these anomalies have been addressed.^17^ The focus is now on long-term risk for CVD rather than solely on 10-year risk for CHD. The new cutoff point for defining ‘high risk’ is a risk of 10% or more of death from any cardiovascular event in the next 10 years (previously it was 20% or more). Other modifications include the use of new risk-stratification scores (the updated Framingham CVD risk profiles and the Reynolds risk score for women).^18^,^19^ New major risk categories are patients with systemic autoimmune collagen vascular disease (such as systemic lupus erythematosus and rheumatoid arthritis) as these disorders are known to be associated with a significantly increased relative risk for CVD.^20^ Women with a history of pre-eclampsia, gestational DM or pregnancyinduced hypertension are also deemed to be at major risk.^21^,^22^

When assessing risk, it is advised that the use of CVD risk biomarkers (such as ultra-sensitive C-reactive protein) and imaging technologies (such as coronary calcium-scoring assessment and carotid intima–media thickness) be reserved for refining risk estimates in patients where there is uncertainty about the need to start statin therapy.^23^,^24^

The 2011 guidelines also focus on stroke and heart failure. As women age, their risk for stroke and HF tends to increase in excess of the risk for CHD.

A new concept of ‘ideal cardiovascular health’ has been proposed and should be adhered to in all women. This is the absence of clinical CVD and the presence of ideal levels of total cholesterol (< 5.2 mmol/l), untreated blood pressure less than 120/80 mmHg, untreated fasting blood glucose less than 5.6 mmol/l, body mass index less than 25 kg/m^2^, and a lifestyle that includes smoking abstinence and physical activity (for adults aged 20 years or more of at least 150 minutes per week of moderate-intensity exercise, and at least 75 minutes per week of vigorous-intensity exercise, or a combination of both). When achieved or maintained into middle age, the overall pattern of ideal cardiovascular health is associated with greater longevity, reduction in risks for CVD events and greater quality of life in older age.^25^

In the new millennium, there is no longer any doubt about what strategies and treatment are required to reduce CVD in women. The major hurdle is to implement these guidelines early. This is particularly so in low- and middle-income countries. If we are to have any impact on the looming pandemic of CVD in the developing world, such as sub-Saharan Africa, we should not hesitate to adopt these updated guidelines.
